# Never Travel Alone: The Crosstalk of Circulating Tumor Cells and the Blood Microenvironment

**DOI:** 10.3390/cells8070714

**Published:** 2019-07-13

**Authors:** Simon Heeke, Baharia Mograbi, Catherine Alix-Panabières, Paul Hofman

**Affiliations:** 1 Université Côte d’Azur, CHU Nice, FHU OncoAge, 06000 Nice, France; 2 Université Côte d’Azur, CNRS UMR7284, Inserm U1081, Institute for Research on Cancer and Aging, Nice (IRCAN), FHU OncoAge, 06000 Nice, France; 3 Laboratory of Rare Human Circulating Cells (LCCRH), University Medical Centre, EA2415, Montpellier University, 34093 Montpellier, France; 4 Laboratory of Clinical and Experimental Pathology and Biobank BB-0033-00025, Pasteur Hospital, FHU OncoAge, 06000 Nice, France

**Keywords:** circulating tumor cells, hematological cells, neutrophils, platelets, liquid biopsy

## Abstract

Commonly, circulating tumor cells (CTCs) are described as source of metastasis in cancer patients. However, in this process cancer cells of the primary tumor site need to survive the physical and biological challenges in the blood stream before leaving the circulation to become the seed of a new metastatic site in distant parenchyma. Most of the CTCs released in the blood stream will not resist those challenges and will consequently fail to induce metastasis. A few of them, however, interact closely with other blood cells, such as neutrophils, platelets, and/or macrophages to survive in the blood stream. Recent studies demonstrated that the interaction and modulation of the blood microenvironment by CTCs is pivotal for the development of new metastasis, making it an interesting target for potential novel treatment strategies. This review will discuss the recent research on the processes in the blood microenvironment with CTCs and will outline currently investigated treatment strategies.

## 1. Introduction

Circulating tumor cells (CTCs) have been extensively studied over the last decades, in particular as they play a crucial role in the diagnosis and the prognosis in many solid tumors as well as due to their predictive value associated with cancer targeted therapies as well as with immunotherapies [1,2,3]. CTCs are present in the blood stream as isolated CTCs (iCTCs) or in clusters of variable sizes that are often referred to as circulating tumor microemboli (CTMs) [4]. Following their migration from the primary site of the tumor into the blood, the tumor cells are constrained to high pressure and turbulences due to the blood stream and have to develop mechanisms of resistance for survival to consequently be able to adhere to the endothelium for tissue invasion and development of metastases [5]. Moreover, some CTCs are also able to come back to the primary tumor site and, consequently, to participate to the tumor growth [6]. However, the physical characteristics allowing the CTCs to survive are only partially known. Nevertheless, the biological characteristics of these cells and the phenotypic, genetic, and epigenetic modifications occurring during their migration from the primary tumor site until the development of distant metastases are beginning to be unraveled.

CTCs need to undergo significant changes to survive in the bloodstream—a new different environment. Thus, CTCs are challenged by physical forces in the circulation, they have to avoid being detected and killed by the immune system and finally, they need to extravasate from the blood stream to become the seed of new metastatic site(s) [7]. Recent works demonstrated that most of CTCs are not single cells travelling the blood alone but are accompanied by a plethora of blood cells and other CTCs and that a close interaction in the blood microenvironment is certainly needed to establish novel metastasis [8]. Interfering with this new microenvironment might help to develop strategies reducing the metastatic potential of tumors [8].

The aim of this review is therefore to summarize current knowledge concerning the role of the blood microenvironment and the different biological mechanisms occurring during its cross talk with CTCs. Additionally, potential therapeutic strategies and clinical approaches are discussed.

## 2. Brief Background on the Pathophysiology of CTCs Into the Blood Stream

### 2.1. The CTCs and the Constraints Due to the Blood Circulation

CTCs derive from primary tumor and/or metastatic sites and are consequently not adapted to the manifold challenges in the blood stream. Importantly, the flow of the blood stream, especially when passing the heart chambers, exposes cells to high mechanical sheer forces that can either directly destroy non-adapted cells or induce apoptosis in them [5,9,10]. Interestingly, CTCs seem to be stiffer than blood cells demonstrating their low adaptation to the blood stream [11] and tumor cells seem to be sensitive to those sheer forces indicating that the majority of CTCs will undergo apoptosis rather than forming metastasis in patients [12]. However, the different hemodynamic forces are important to allow the extravasation of tumor cells as they also remodel the endothelium [13], and consequently more knowledge on the biophysical properties allowing the formation of metastasis are needed [11]. Additionally, CTCs are directly exposed to the immune system and consequently they need to evade the detection from immune cells. Interestingly, programmed death ligand 1 (PD-L1), a costimulatory molecule inhibiting immune response can be expressed on CTCs and is associated with worse prognosis in lung [14,15] and head and neck cancer patients [16]. This indicates the active modulation of the immune response of CTCs to survive in the blood stream.

Lastly, as CTCs from cancers are of epithelial origin, they are adapted to grow in a network with other cells and are tightly interconnected by transmembrane proteins called integrins [17,18]. CTCs that leave the primary tumor site and enter the bloodstream lose the tight interaction with the surrounding cells, which can induce apoptosis in those cells through a phenomenon called anoikis [19]. Consequently, suppression of anoikis is required for survival of CTCs in the bloodstream [20], either by interaction of CTCs with other blood cells or by internally suppressing anoikis by activation of integrin signaling independent of cell–cell contacts [21].

### 2.2. Isolated CTC and Circulating Tumor Microemboli

While single CTCs are travelling in the blood stream, it has been demonstrated that CTC clusters or circulating tumor microemboli (CTMs) have a dramatically increased metastatic potential, as demonstrated in lung [4] and breast cancers [22]. Interestingly, a recent study conducted in 43 breast cancer patients demonstrated that CTC clustering alters DNA methylation patterns and increases stemness and consequently metastasis [23]. Single-cell bisulfite sequencing of single CTCs and CTCs derived from clusters revealed that transcription factors that are associated with a stem cell-like phenotype, like OCT4, NANOG, or SOX2, where hypomethylated in CTC clusters compared to single CTCs [23]. Interestingly, the authors also performed a drug-screening using 2486 FDA approved drugs to analyze their ability to interfere with CTC clustering. Thirty-one drugs have been detected that could serve as novel treatment to reduce the metastatic potential in breast cancer patients [23]. Consequently, the metastatic potential of CTCs might be limited in isolated CTCs and more research focusing on CTC clusters (CTMs) rather than on single cells might allow the design of novel treatment strategies to interact with the formation of CTC cluster to avoid metastasis in cancer patients.

## 3. Interaction of CTCs with Neutrophils

**Figure 1:**
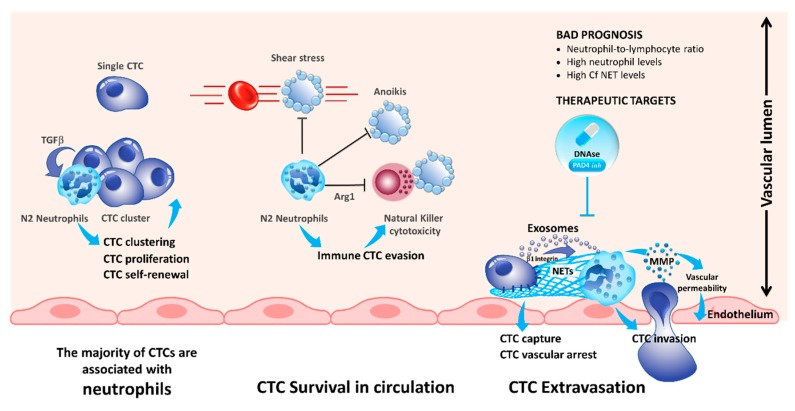
Compelling evidence indicates that blood neutrophils can offer proliferative and survival advantages to circulating tumor cells (CTCs) during their journey in the blood stream, rendering them more competent for metastasis development. Tumor-derived inflammatory factors strongly stimulate neutrophils to extrude chromatin webs called “neutrophil extracellular traps” (NETs). NETs, in turn, provide a niche to CTCs, arrest CTCs rolling, and promote metastasis. As such, understanding interaction of inflammatory N2 neutrophils with CTCs provides new potential therapeutic targets for disrupting these deadly metastatic seeds. As an example, blockade of NET formation using peptidylarginine deiminase 4 (PAD4) pharmacologic inhibitor or DNAse may decrease CTC colonization.

The role of neutrophils in cancer progression has been extensively studied recently [24,25]. Previously, it has been demonstrated that increased levels of circulating neutrophils are associated with bad prognosis in advanced cancer patients [26,27]. Moreover, the neutrophil-to-lymphocyte ratio has been demonstrated to be a prognostic factor in solid tumors [28]. In a recent study, Szczerba et al. demonstrated that within white blood cells (WBCs)–CTCs clusters of breast carcinoma, CTCs are associated with neutrophils in the majority of the cases [29]. Interestingly, using single-cell RNA sequencing, the authors showed that the transcriptome profiles of CTCs associated with neutrophils are different from those of CTCs alone, with differentially expressed genes that outline cell cycle progression leading to a more efficient metastasis formation. The authors noted that WBC–CTC clusters are relatively rare (less than 3.5%), whereas iCTCs alone are present in 88% of cases and CTCs clusters in less than 9% of the cases [29]. Despite, neutrophils directly interact with CTCs via ICAM-1 and neutrophils bound to CTCs facilitate the interaction of CTCs with endothelial cells in the liver, thereby promoting extravasation and liver metastasis [30]. Consequently, neutrophils play a major role for the CTC extravasation across the endothelial barrier and the onset of metastases (Figure 1) [31].

Neutrophils are able to generate neutrophil extracellular traps (NETs) by secreting their chromatin content during a process known as NETosis [32]. Initially, this process was described to be a mechanism to kill bacteria [33]. However, recent studies demonstrated that NETs are also promoting metastasis across various cancers [34,35,36,37,38]. Tohme et al. demonstrated that the NET formation induces a TLR-9 mediated response in cancer cells, which increased the migration and proliferation of CTCs [37]. Interestingly, it was shown that NETs promote extravasation of CTCs but in an IL-8 dependent manner. Consequently, blocking of IL-8 reduced the extravasation of tumor cells and neutrophils (Figure 1) [31]. Additionally, tumor-derived exosomes were also able to induce NET formation in neutrophils isolated from mice treated with granulocyte colony-stimulating factor (G-CSF) [39]. This phenomenon is even more important for tumors producing a large quantity of G-CSF associated with a high number of blood neutrophils [40,41]. Mechanistically, it has been demonstrated that the interaction of CTCs with NETs is mediated by β1-integrins expressed on tumor cells [42]. It is noteworthy that this integrin is physiologically overexpressed during infections and sepsis [42]. Generally, resolving NETs, for example, using DNAse I administration, reduced the number of metastasis making the NETs an interesting target for novel treatments reducing metastasis in patients (Figure 1) [34,35,36,37,38].

Different populations of neutrophils have different tumor promoting effects, and neutrophils are heavily modulated during cancer progression [43,44]. Consequently, extensive research is necessary to better decipher the role of the interaction of neutrophils and CTCs.

## 4. Interaction of CTCs with Myeloid-Derived Suppressor Cells

Myeloid-derived suppressor cells (MDSCs) are a heterogenous group of cells that are derived from the bone marrow and that are able to suppress the immune response via the suppression of T-cell response [45]. Commonly, MDSCs are classified in polymorphonuclear MDSCs (PMN-MDSC) and monocytic MDSCs (M-MDSC) [45]. As MDSCs are enriched in tumor tissue and able to suppress the immune system, it has been proposed that the interaction of CTCs with MDSCs might also promote metastasis [46]. Indeed, heterogenic clusters of CTCs and MDSCs have been reported in melanoma, pancreatic, and breast cancer patients [47,48], and co-culture of MDSCs with CTCs and T-cells demonstrated the T-cell suppressive effect of MDSCs [48]. Moreover, CTCs and MDSCs interact directly with each other, and increased reactive oxygen species (ROS) production in MDSCs induced NOTCH1 in CTCs, hereby promoting CTC proliferation [47]. Consequently, blocking the MDSC–CTC interaction might inhibit CTC proliferation and CTC immune evasion and might be an interesting target in anti-cancer therapy.

## 5. Interaction of CTCs with Platelets

**Figure 2:**
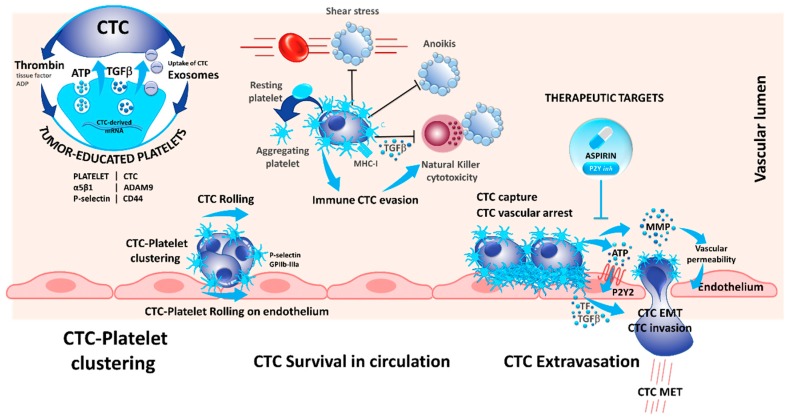
The dialogue between platelets and CTCs is reciprocal: CTCs activate and educate platelets while platelets contribute to CTCs’ survival, escape from immune surveillance, tumor–endothelium interactions, and dissemination. Secretion of α-granules by activated platelets release high levels of TGF-β and ATP, a powerful activator of epithelial-to-mesenchymal transitioned (EMT) state and an endothelium relaxation factor (via P2Y), respectively. Inhibition of platelet α-granules secretion (Cox1) by aspirin, or of P2Y may abolish the metastatic potential of CTCs.

In 1973, the role of platelets in cancer metastasis was already described, and the following work highlighted the role of platelets in cancer progression, especially during cancer metastases [49]. Indeed, different mechanisms occur during platelet–cancer cell interactions and crosstalk: (i) cancer cells can induce platelet activation; (ii) platelets support cancer metastasis and enhance cancer cell adhesion and arrest in vasculature; (iii) platelets assist immune evasion of cancer cells, and finally, (iv) platelets can enhance cancer evasion and tumor angiogenesis (Figure 2).

Interestingly, platelets can take up circulating mRNA from the CTCs, suggesting a possible modification in the platelet transcriptome that resembles the tumor profile [50]. In this context, platelets that circulate through and contact tumor sites can undergo modification due to the sequestration of RNA and biomolecules, which led to the concept of tumor-educated platelets (TEP) and may serve as an informative tool in cancer diagnosis [51,52]. This adherence could also help to decrease the impact of the pressure and the turbulence, especially in the heart chamber on the CTCs and can protect the CTCs against the physical stress in the blood stream (Figure 2) [53]. Indeed, platelets can form aggregates with CTCs, and CTCs induce platelet aggregation in a process known as tumor-cell-induced platelet aggregation (Figure 2) [54].

Platelets interact with tumor cells during blood dissemination leading to platelet activation and release of soluble mediators that alter the phenotype of the tumor cells and surrounding host cells [55]. However, the proximal events that initiate platelet activation are only partially characterized. It has been recently demonstrated that CD97 expressed on tumor cells may be involved in platelets activation [56]. CD97 is a g-protein coupled receptor that is undetectable in normal tissues except for smooth muscle cells but is abnormally expressed in different types of solid tumors. Ward et al. demonstrated that CD97 is able to activate platelets, which in turn secrete several mediators of the endothelial barrier, including ATP, which promotes evasion of CTCs off the blood stream and consequently promotes metastasis [56]. Additionally, the platelet P-selectin interacts with tumor CD44, and the fibrinogen receptor GPIIb-IIIa are involved in platelet rolling on CTCs and in platelet–CTC emboli (Figure 2) [57,58]. This leads to several alterations of platelets including protein synthesis, exosome release, blebbing of the membrane, and splicing of mRNAs [59]. Therefore, it seems that platelets form homotypic aggregates at the center of clusters that are surrounded by tumor cells at the periphery [54].

Despite the alterations induced by a direct contact between platelets and CTCs, the production of cytokines by platelets modifies the phenotype of CTCs. Molecular mechanisms by which CTCs maintain an epithelial-to-mesenchymal transitioned (EMT) state remain unclear. CTC clusters isolated from patients with advanced breast cancers highly exhibit mesenchymal markers and show an abundance of attached CD61-positive/platelets [60]. TGFβ1 secretion by alpha granules induces or increases the EMT observed in CTCs [55]. Likewise, platelets increase the tissue factor (TF) and the P2Y12 receptor activity, and both participate in EMT [61,62]. Moreover, platelets are involved in the adherence of CTCs to the endothelial barrier and to the transmigration of CTCs into the tissue for the development of metastasis [63]. One of the receptor ligand pair identified with such function is the ADAMA9 on CTCs that binds to the integrin α5β1 on the surface of platelets. This interaction is believed to promote platelet activation, granule secretion, and the transmigration of tumor cells through the endothelium [64]. Other mechanisms arising during the interaction between platelets and CTCs promote the migration of CTCs across the vasculature barrier: CTC-induced platelet aggregation leads to the release of ATP stored in dense granules; released ATP binds to the P2Y2 receptor stimulating cancer cell intravasation and metastatic dissemination [65]. Additionally, platelets and megacaryocytes play a major role in the survival of CTCs in the blood stream by different mechanisms. Platelets can protect certain CTCs against anoikis (a form of apoptosis that is induced when adherent cell lose contact to the surrounding cells) [66]. Even more, the adherence of platelets at the surface of CTCs may protect the CTCs to be recognized by some circulating immune cells, thereby promoting cell survival (Figure 2) [59,67]. Interestingly, platelets exert paracrine suppression of NK-mediated cytolytic activity. TGFβ released from activated platelets counteracts NK granule mobilization, cytotoxicity, and interferon-γ secretion [68]. Besides, platelet–CTC interaction can lead to the transfer of platelet major histocompatibility complex class I (MHC-I) to tumor cells preventing NK cell recognition via direct cell contacts [69]. This phenomenon is complex and not completely understood, and the platelets need to be activated at contact of CTCs entering in the circulation. Therefore, CTCs can release thrombin that attracts, activates, and aggregates platelets on their surface [54,59,70]. Several factors such as TF, thrombin, and ATP secreted by either platelets or CTCs induce platelets activation and formation of platelet–cancer cell aggregates [54,70].

Due to their multifaceted role in cancer metastasis, blocking of platelet–CTC interaction has also been studied as pharmacological target to reduce metastasis. Recently, Gareau et al. demonstrated that blocking this interaction using the P2Y12 inhibitor ticagrelor, reduced the number of metastasis and prolonged survival in a murine breast cancer model [71]. Additionally, in a clinical phase II study investigating the effect of aspirin on CTCs, less CTCs were detected in colorectal cancer patients upon aspirin treatment and the detected CTCs showed a more epithelial phenotype. Unfortunately, the results were not confirmed in a breast cancer model [72]. However, both aspirin and P2Y12 inhibitors inhibit platelet activation and demonstrate that modulation of platelets can reduce CTCs and metastasis, and the recent trials paved the way to actively investigate how the modulation of platelets can prevent metastasis-related cancer death [71,72].

## 6. Interaction of CTCs with Macrophages

Tumor-associated macrophages (TAMs) play a key role in activating dissemination and providing protection against the immune system [73]. However, the interplay of macrophages and CTCs is poorly understood. Previous works have been made to investigate the interaction between the macrophages and the CTCs in small cell lung carcinoma (SCLC) [74,75]. In these latter studies, different interactions have been observed by establishing SCLC cell lines and co-culture experiments with peripheral blood mononuclear cells (PBMCs). The authors showed that interaction of PBMCs with SCLC cells promote the differentiation of monocytes into macrophages, which express CD14, CD163, and CD68. These macrophages can secrete different cytokines such as osteopontin, monocyte chemoattractant protein-1, IL-8, chitinase 3-like 1, platelet factor, IL-1ra, and the matrix metalloproteinase-9 [74]. Likewise, PCa prostate cancer cells cultured with monocyte conditioned cell culture media, showed an increased invasion in vitro mediated by the IL-13Rα2 receptor expressed on cancer cells [76]. Additionally, Wei et al. demonstrated that the crosstalk of macrophages with tumor cells is necessary for the induction of EMT and release of CTCs into the blood stream. In their study, expression of IL6 of TAMs increased the secretion of CCL2 in tumor cells, which in turn recruited new macrophages [77].

The discussed studies demonstrate that the interaction of macrophages and tumor cells is not only important for progression at the primary tumor site but also for the promotion and differentiation of CTCs [77]. However, the interaction might be much closer than previously expected. Zhang et al. demonstrated that some circulating macrophages might be able to phagocyte apoptotic CTCs and incorporate the tumor DNA into their nuclei, consequently obtaining some malignant features like expression of epithelial markers (such as cytokeratins) and stem cell markers (e.g., OCT4) [78]. Consequently, circulating monocytes from solid cancer patients can express both CD163 and EpCAM. This led to the concept of “tumacrophages,” which have the potential of invasive tumor cells but are protected against the immune system [78]. Even more, Gast et al. showed in a seminal work that viable tumor cells can fuse with macrophages to create hybrid cells, sharing markers of both tumor cells and macrophages [79]. Hybrid cells sharing epithelial (EpCAM expression) and hematological cell markers (CD45) were protected from detection by the immune system and correlated with disease stage and overall survival across several cancers [79]. Similar results were recently confirmed in a glioblastoma model where tumor cell–macrophage fusion cells demonstrated an increased invasive potential [80].

Consequently, the interplay of CTCs with macrophages is certainly important for metastasis and the discovery of tumor cell–macrophage fusion cells will help to develop novel biomarkers for cancer progression as well as novel potential therapeutic targets to block metastasis in patients.

## 7. Interaction of CTCs with Lymphocytes

Tumor cells constantly need to avoid being detected by immune cells to avoid being killed by them [81]. Likewise, CTCs in the blood stream are constantly required to avoid activation of the immune cells. The recent success of anti-cancer immunotherapy, most notably of checkpoint blocking antibodies, in several cancers have demonstrated that the immune system can be reactivated to target cancer cells [82]. Unfortunately, only limited studies have been carried out on the interaction of CTCs and lymphocytes. However, an inverse correlation between CD3^+^, CD4^+^, and CD8^+^ peripheral T-lymphocytes and CTCs in NSCLC [83] and between CD8^+^ peripheral lymphocytes in breast cancer [84] have been shown. Moreover, several studies demonstrated that regulatory T-cells infiltrating the tumor or detected in the peripheral blood are significantly more prevalent in breast cancer patients with CTCs than in patients without detectable CTCs [83,84,85]. While unfortunately mechanistic studies evaluating the interplay between T-lymphocytes and CTCs are lacking, the present studies, however, indicate that immune suppression by regulatory T-cells help in tumor cell dissemination in the blood stream. However, the responsible targets and mechanisms have to be unraveled to better understand the interplay of CTCs with lymphocytes.

Additionally, CTCs seem to be able to block interaction with lymphocytes by upregulating the programmed death ligand 1 (PD-L1) that inhibits the activation of T-lymphocytes [2]. This allows the CTCs to avoid being detected by the immune system and was indeed correlated with worse prognosis in NSCLC patients undergoing radio (chemo)-therapy [86]. Nevertheless, this might be only one of many mechanisms CTCs have to adapt to avoid detection by immune cells in the blood stream, and further research and clarification is needed.

## 8. Challenges and Perspectives

While CTCs have long been considered to be isolated cells floating in the blood stream, recent research demonstrated the close interaction of CTCs with the blood microenvironment. CTCs need to establish close interaction not only with platelets and neutrophils, but also with macrophages and endothelial cells to resist the physical stress in the blood stream and to evade detection by the immune system to finally leave the blood stream to establish new metastatic sites (Table 1). 

While seminal research demonstrated well that the blood microenvironment is crucial for cell seeding, the mechanisms and interaction networks are not fully understood, and more research is needed. However, blocking the interaction of CTCs with platelets [71,72] as well as the resolving of NETs [34,35,36,37,38] demonstrated that targeting the interaction of CTCs with other cells is a promising therapeutic target and future research will certainly establish novel treatments to improve survival in cancer patients.

## Figures and Tables

**Table 1:** Summary of interactions of circulating tumor cells with other cell types in the blood microenvironment.

Interaction of CTCs with Other Cell Type	Interacting Targets/Processes	Effect	References
Neutrophils	ICAM-1	Facilitating interaction with endothelial cells and consequently extravasion off the blood stream.	[30]
	β1-integrin, tumor-derived exosomes	Formation of neutrophil extracellular traps (NETs) promoting proliferation and extravasion.	[31,37,39,42]
Myeloid-derived suppressor cells (MDSCs)	Reactive oxygen species (ROS) production by MDSCs	Increased proliferation of CTCs and inhibition of T-cells.	[47,48]
Blood platelets	Exosomes	Formation of tumor-educated platelets (TEPs).	[50,51,52]
	CD97, CD44, ADAMA9-α5β1 integrin, ATP	Modulation of endothelial cells by platelets leading to extravasion of CTCs.	[56,63,64,65]
	Cytokines produced by platelets	Induction of epithelial-to-mesenchymal transition in CTCs.	[55,60,61,62]
	TGFβ secreted by platelets	Suppression of cytolytic NK cells.	[68]
Macrophages	Cytokines produced by Macrophages	Increased invasion EMT of CTCs and immune suppression.	[74,76,77]
	Fusion with CTCs	Formation of “tumacrophages” that are protected from immune detection with invasive potential.	[78,79,80]
Lymphocytes	PD-L1	Suppression of cytotoxic T-cells.	[2]
